# Understanding health education needs of pregnant women in China during public health emergencies: a qualitative study amidst the COVID-19 pandemic

**DOI:** 10.3389/fpubh.2024.1271327

**Published:** 2024-05-02

**Authors:** Xiaojuan Su, Yuezhen Zhang, Meide Chen, Xiangyang Xu, Guihua Liu

**Affiliations:** ^1^Department of Nursing, Quanzhou Medical College, Quanzhou, Fujian, China; ^2^Nursing Department, Quanzhou Women and Children's Hospital, Quanzhou, Fujian, China; ^3^Department of Imaging, Anxi County Hospital, Quanzhou, Fujian, China; ^4^Department of Child Health Care, Fujian Maternity and Child Health Hospital College of Clinical Medicine for Obstetrics and Gynecology and Pediatrics, Fujian Medical University, Fuzhou, Fujian, China

**Keywords:** pregnant women, health education, public health emergencies, qualitative research, COVID-19

## Abstract

**Background:**

Public health emergencies impose unique challenges on pregnant women, affecting their physiological, psychological, and social wellbeing. This study, focusing on the context of the corona virus disease in 2019 (COVID-19) pandemic in China, aims to comprehensively explore the experiences of pregnant women amidst diverse public health crises. Herein, we investigate the health education needs of pregnant Chinese women in regard to public health emergencies to provide a scientific foundation for the development of targeted health education strategies.

**Objective:**

The study described in this article aims to explore the health education needs of pregnant Chinese women in the context of public health emergencies specifying the types of emergencies of pandemics and to provide a scientific basis for targeted health education interventions.

**Methods:**

Thirteen pregnant women were purposively selected, and the rationale for this sample size lies in the qualitative nature of the study, seeking in-depth insights rather than generalizability. Data collection involved semi-structured interviews, and the Colaizzi, which is a structured qualitative technique used to extract, interpret, and organize significant statements from participant descriptions into themes, providing a comprehensive understanding of their lived experiences.

**Results:**

The analysis yielded six prominent themes encompassing the following areas: I. Personal protection and vaccine safety; II. Knowledge of maternal health; III. Knowledge of fetal health; IV. Knowledge of childbirth; V. Knowledge of postpartum recovery; and VI. Knowledge sources of health education for pregnant women and their expectations of healthcare providers. Theme I was analyzed with two sub-themes (needs for personal protection knowledge, vaccine safety knowledge needs); Theme II was analyzed with three sub-themes (nutrition and diet, exercise and rest, sexual life); Theme III was analyzed with three sub-themes (medications and hazardous substances, pregnancy check-ups, and fetal movement monitoring); Theme IV was analyzed with three sub-themes (family accompaniment, analgesia in childbirth, and choice of mode of delivery); Theme V was analyzed with one sub-theme (knowledge of postnatal recovery); Theme VI was analyzed with one sub-theme (expectations of Healthcare providers). Sub-themes within each main theme were identified, offering a nuanced understanding of the multifaceted challenges faced by pregnant women during public health emergencies. The interrelation between sub-themes and main themes contributes to a holistic portrayal of their experiences.

**Conclusion:**

The study emphasizes the need for healthcare professionals to tailor health education for pregnant women during emergencies, highlighting the role of the Internet in improving information dissemination. It recommends actionable strategies for effective health communication, ensuring these women receive comprehensive support through digital platforms for better health outcomes during public health crises.

## 1 Introduction

Pregnancy ushers in a period of diverse physiological, psychological, and social changes, necessitating specialized attention to address the unique needs of expectant women (1). The coronavirus disease outbreak in 2019 (COVID-19) has disproportionately affected pregnant individuals worldwide, with altered immune responses and compromised lung function placing them at heightened risk of infection (2). This increased susceptibility raises concerns about the potential for adverse pregnancy outcomes (3). In response to the COVID-19 pandemic, home isolation emerged as an important preventive measure against the spread of the virus, presenting pregnant women with the dual challenge of managing the inherent health challenges of pregnancy alongside the additional stresses and complexities introduced by the pandemic (4).

In this evolving landscape, the content and methods of delivering health education to pregnant women have significantly changed. Despite these developments, research on healthcare for pregnant women during epidemics, particularly within the Chinese context, remains scarce. Existing literature has largely focused on the psychological impacts of such crises on pregnant women (5–9).

In addition to focusing on the psychological wellbeing of pregnant women, it is also important to consider the broader spectrum of challenges they face, including physical health concerns and the need for social support during epidemics. Studies have demonstrated that epidemics like COVID-19 pose significant risks to the physical health of pregnant women, increasing the likelihood of complications such as preterm birth, preeclampsia, and gestational diabetes (10, 11). Moreover, the impact of social distancing and healthcare system strain on the availability and quality of prenatal care further complicates these physical health challenges. In addition, social support plays a vital role in mitigating the psychological impact of epidemics on pregnant women (12). The reduction in face-to-face interactions and the increased reliance on virtual platforms for support have transformed the nature of social networks, with varying implications for maternal wellbeing. By integrating this broader spectrum of research, the complex interplay between psychological, physical and social factors affecting pregnant women during epidemics can be more clearly determined. This comprehensive approach not only enriches our understanding of their health education needs but also highlights the necessity for multifaceted health education strategies that address these interconnected aspects.

This study aims to fill this research gap by utilizing qualitative interviews to investigate the health education needs of pregnant Chinese women during public health emergencies, with a specific focus on the COVID-19 outbreak that began in China in 2021. The focus on China in this study is particularly relevant due to the country's unique challenges during the COVID-19 pandemic. China was the epicenter of the COVID-19 outbreak, facing unprecedented public health challenges that required rapid and large-scale interventions (13). This situation significantly impacted pregnant women, who faced heightened risks due to altered immune responses and compromised lung function, increasing their vulnerability to infection. Furthermore, the implementation of stringent lockdown measures and hospital visitation restrictions introduced additional complexities for accessing prenatal care and support systems (14, 15). These factors underscore the importance of exploring the health education needs of pregnant Chinese women during such emergencies. By concentrating on the Chinese context, this study aims to uncover insights that can inform targeted health education strategies, acknowledging the nuanced ways in which cultural, social, and healthcare system factors intersect to influence health education needs and outcomes during public health crises.

Thus, we designed this present study to explore the specific health education requirements and information-seeking behaviors of pregnant Chinese women during the COVID-19 outbreak. The ultimate goal is to provide insights for healthcare institutions and professionals to develop effective health education strategies. These strategies are intended to safeguard the health and wellbeing of expectant mothers and their unborn children, offering a foundational framework to guide future research and practice in health education during similar health crises.

## 2 Research methodology

### 2.1 Study participants

A purposive sampling method was utilized to select 13 pregnant women for pre-interview participation who attended pregnancy healthcare examinations at an obstetrics outpatient clinic in a maternity and child healthcare hospital in Quanzhou City, Fujian Province, from December 2021 to June 2022. The selection process adhered to specific inclusion criteria: (1) Pregnant women aged 35 years or younger, with a gestational age of 12 weeks or more; (2) No presence of comorbidities or complications at the time of study; (3) A minimum education level of junior high school, ensuring adequate literacy for understanding and responding to the study's requirements; and (4) Willingness to provide informed consent. The study exclusion criteria were: (1) Severe audiovisual or cognitive impairments that could affect the participant's ability to understand questions or provide coherent responses; (2) A history of mental illness; and (3) Unwillingness to cooperate after the study's objectives and procedures had been explained by the investigator.

The study sample size was determined by the principle of information saturation, where the recruitment continued until no new themes were identified in the data. Following these criteria, 13 pregnant women were selected for the study, each assigned a unique identifier ranging from A to M.

### 2.2 Research instruments

#### 2.2.1 Basic information questionnaire

This questionnaire was designed to collect baseline data from the pregnant women participating in the study, such as their gestational age, personal age, level of education, pregnancy type, and whether the pregnancy was intentional.

#### 2.2.2 Interview outline

The interview outline was initially drafted by referring to the relevant literature (16–20) and by consultations with experts in nursing, obstetrics, and individuals outside the participant pool for pre-testing. After undergoing two rounds of revisions by nursing and obstetric experts and three pre-test sessions with non-participants, the final interview guide was established. The outline covered the following key questions:

(1) During the COVID-19 epidemic, what are your specific needs for health education, information, or healthcare services related to pregnancy?
(2) What are the primary methods or channels through which you seek out health education or information on pregnancy?
(3) How do you prefer to receive pregnancy-related health education and information from healthcare providers?

### 2.3 Research methods

#### 2.3.1 Data collection

The study was approved by the Ethics Committee of Quanzhou Medical College (Approval No. 2021007). Using the phenomenological method, this qualitative research conducted in-depth, face-to-face interviews with each participant, lasting between 20 and 40 min. To ensure the accuracy and completeness of the data, follow-up telephone calls were made after each interview for content confirmation, allowing participants to add further insights or clarify previous statements.

Prior to conducting the interviews, necessary preparations were made, including the setup of tape recorders and notebooks to accurately capture the dialogue. Participants were informed about the study's objectives, its importance, the confidentiality of their responses, and their rights as participants, establishing a foundation of trust. Interviews were held in a private obstetrics room to guarantee confidentiality and create a comfortable setting for the participants.

To uphold the integrity of our study and minimize potential biases in data collection, rigorous steps were taken to train the interviewers and ensure their neutrality throughout the interviewing process. The following measures were implemented: (1) Interviewer training: prior to engaging with participants, interviewers underwent comprehensive training sessions conducted by experienced researchers in qualitative data collection. These sessions focused on the ethical considerations of interviewing pregnant women during public health emergencies, emphasizing the importance of neutrality, empathy, and open-mindedness. (2) Practice interviews: to refine their interviewing skills and familiarize themselves with potential challenges, interviewers conducted practice interviews with individuals who did not participate in the main study. Feedback from these practice sessions was critically reviewed, and additional training was provided where necessary to enhance the interviewers' proficiency. (3) Use of structured interview guides: interviewers utilized structured interview guides that were carefully designed and reviewed by the research team. These guides aimed to maintain consistency across interviews while allowing for flexibility to explore emergent themes. The questions were framed to minimize any inadvertent biases and to encourage participants to express their experiences openly. (4) Ongoing supervision and debriefing: throughout the data collection period, regular supervision meetings and debriefing sessions were conducted with the interviewers. This provided a platform to discuss any challenges faced during interviews and to address any potential biases that might have emerged. Consistent communication ensured that the research team remained vigilant in maintaining data collection standards.

Utilizing a semi-structured interview format based on a pre-developed outline allowed for the flexibility to adjust questions and their sequence as needed, tailoring the approach to each participant's responses. The researchers actively engaged with participants, guiding the conversation, posing questions, and seeking clarification when necessary, all the while maintaining a focus on the study's central theme.

A key aspect of the methodology was the emphasis on creating an environment conducive to open and honest communication. Participants were encouraged to share their thoughts and feelings freely. In addition to verbal responses, researchers also observed and noted non-verbal cues such as facial expressions, tone, pauses in speech, and body language. These observations were recorded and included in the data analysis to provide a comprehensive understanding of the participants' experiences.

#### 2.3.2 Data organization

After each interview, we stored the original audio data and the general information questionnaire of the research subjects together in a designated folder. Within 2 days of concluding each interview, the audio data was transcribed into textual form and coded according to the alphabetical numbers assigned to each participant (A to M). The time and location of the interview were noted at the beginning of the text. After transcription, the texts underwent a meticulous review process to ensure their accuracy compared to the original recordings. Once validated, these accurate transcripts were saved in the unique digital folder for each participant, facilitating organized data retrieval for analysis.

To enhance the overall credibility of our study, we take the following approach:

Validation of findings: (1) Member checking: to enhance the credibility of our findings, we employed member checking as a form of validation. A subset of participants was provided with a summary of the identified themes and findings. Their feedback and reflections on whether these accurately represented their experiences were actively sought. This iterative process allowed us to refine and validate the interpretation of our data. (2) Peer debriefing: regular debriefing sessions were conducted within the research team. The interdisciplinary nature of our team facilitated a comprehensive discussion of the emerging themes, interpretations, and any potential biases. Peer debriefing served as a form of internal validation, ensuring that diverse perspectives were considered during the analysis. (3) Audit trail: an extensive audit trail was maintained throughout the research process. Detailed records of the data collection, coding, and analytical decisions were documented. This audit trail serves as a transparent account of our research journey, enabling others to follow our analytical processes and decisions.
Triangulation of findings: (1) Data source triangulation: we collected data through multiple sources, including individual interviews and, where applicable, relevant documents. Triangulating data from various sources enriched the depth and comprehensiveness of our findings, providing a more holistic understanding of pregnant women's experiences during public health emergencies. (2) Methodological triangulation: in addition to Colaizzi's phenomenological method, which served as our primary analytical approach, we applied methodological triangulation by exploring quantitative data related to demographic variables. This combination of qualitative and quantitative methods added layers to our understanding and strengthened the robustness of our findings.

## 3 Results

### 3.1 Basic information

The baseline information of the study participants is shown in Table 1.

**Table 1:** Basic information of pregnant women (*n* = 13).

**Number**	**Week of pregnancy (weeks)**	**Age (years)**	**Academic qualifications**	**Maternity**	**Whether to plan a pregnancy**
A	17	30	Master's Degree	Pregnancy 1 birth 0	No
B	22	32	Undergraduate	Pregnancy 2 delivery 1	No
C	32	29	College	Pregnancy 1 birth 0	Yes
D	28	24	College	Pregnancy 1 birth 0	No
E	14	34	High School	Three pregnancies and 2 births	No
F	30	29	Undergraduate	Pregnancy 1 birth 0	No
G	30	34	Graduate Students	Pregnancy 1 birth 0	Yes
H	16	35	Secondary School	Three pregnancies and 2 births	Yes
I	36	31	Secondary School	Pregnancy 2 delivery 1	No
J	38	25	High School	Pregnancy 1 birth 0	No
K	33	28	Secondary School	Pregnancy 1 birth 0	Yes
L	26	25	College	Pregnancy 1 birth 0	No
M	20	27	Secondary School	Pregnancy 1 birth 0	No

### 3.2 Findings

#### 3.2.1 Theme 1: personal protection and vaccine safety

##### 3.2.1.1 Needs for personal protection knowledge

Twelve of the study participants reported concerns about their knowledge regarding personal protective measures in the context of the epidemic. These individuals participated in regular maternity examinations and adopted a range of protective strategies to mitigate the risk of viral infection. Nonetheless, there was prevalent anxiety concerning the potential implications of maternal infection on both fetal and maternal health. While some adhered rigorously to prescribed protective guidelines, they further expressed a desire for healthcare facilities to strengthen their infection control protocols. Conversely, a subset of participants decided against attending hospital-based maternity check-ups, citing these concerns.

Participant A detailed the protective measures she adopted, saying, “For my pregnancy test last week, I wore two masks and goggles. Upon returning home, I immediately used alcohol for disinfection, changed, and thoroughly cleaned everything before going back to the hospital. My aim is to ensure complete protection and prevent any accidental virus transmission,” she added, visibly concerned (frowns).

Participant B expressed apprehension about hospital environments: “I avoid hospital visits unless absolutely necessary for my baby's health. Hospitals are crowded, and surfaces like door handles and elevator buttons might not be sanitized properly. I wish there were more rigorous disinfection practices in place.”

Participant C shared her unease about hospital visits, stating, “Even though I try to keep a distance, people often crowd together in lines. The hospital's lax approach to managing crowds increases my anxiety about obtaining a safe pregnancy test,” she remarked, frowning (frowns).

Participant D considered alternative preventive measures: “I've looked into mask effectiveness online and thought about herbal remedies for COVID-19 prevention. A neighbor knowledgeable in traditional medicine suggested a formula I'm thinking of trying.”

Participant E, recognizing the pandemic's severity, said, “With the current state of the COVID-19 epidemic, it's crucial for me to learn about protective measures during pregnancy and how to manage risks.”

Participant G, voicing her fears, mentioned, “The possibility of COVID-19 affecting my baby and me is a significant concern. I'm avoiding crowded places and taking all necessary precautions. I'm also looking for information on preventing infection during pregnancy.”

Participant H stressed the importance of medical advice: “I'm looking for guidance from healthcare professionals on how to protect myself and my fetus during the pandemic.”

During her second pregnancy, Participant H felt more anxious: “This pandemic has made me more worried than during my first pregnancy. I'm concerned about the virus's potential impact on my fetus during childbirth.”

Participant I was confused by conflicting information: “Although I've seen reports on TV and online about COVID-19′s effects on pregnant women and fetuses, I'm still unsure about the best actions to take.”

Approaching her due date, Participant J shared her concerns about hospital visits: “With my due date near, the thought of hospital visits is worrisome. Constantly wearing a mask is challenging, and my anxiety grows daily,” she said, frowning.

Participant K discussed the difficulties in accessing prenatal care: “Every maternity check-up requires health and travel code verification, which is tedious. Despite having appointments, long waits are common. The strict infection control and limited check-up slots often conflict with my schedule, leaving me worried about completing necessary checks for my fetus's health,” she explained, frowning.

Participant L contemplated the repercussions of contracting the virus: “If I were to catch the virus, I'm concerned about its impact on my unborn child, especially if it complicates hospital visits for labor or delivery.”

Expressing fears of reinfection, Participant M said, “Having had COVID-19 before, I'm worried about how it might affect my unborn child and the unsettling thought of getting the virus again,” her concern evident in her expression.

Collectively, while all participants expressed concerns about their knowledge of personal protective measures, their levels of anxiety and the extent of protective strategies they adopted varied significantly. For example, Participant A took extensive protective measures, including wearing multiple masks and goggles, which indicates a high level of anxiety and a strong desire for comprehensive protection. In contrast, other participants, like Participant D, explored alternative preventive measures, such as herbal remedies, suggesting a divergence in trust or confidence in conventional medical advice vs. traditional or alternative health practices.

##### 3.2.1.2 Vaccine safety knowledge needs

Six participants highlighted their need for information on the safety of the COVID-19 vaccine during pregnancy. Concerns were focused on the potential adverse effects on the fetus, particularly when the vaccine was administered during unplanned pregnancies or within the first trimester. Questions also emerged regarding the effectiveness of partial vaccination with only three doses instead of the full series (COVID-19 vaccine full vaccination, meaning that the vaccine should be given in three consecutive doses, with the second dose to be completed as far as possible within 8 weeks of the first dose, and the third dose to be completed as far as possible within 6 months of the first dose).

Participant A shared her concerns about early vaccination: “I was vaccinated with the COVID-19 vaccine early in my pregnancy, which was unexpected. I'm worried about the possible negative effects on my baby's health.”

Participant E, who became pregnant soon after receiving the vaccine, expressed her fears: “I got pregnant 2 weeks after my third dose of the vaccine, without any plans for pregnancy at that time. Had I known, I might have reconsidered getting the vaccine, especially since there were advisories against pregnant women receiving it. The situation is quite worrying now.”

Participant H was confused by the varying advice: “The guidance regarding vaccination for pregnant women appears inconsistent. It's sometimes advised against, yet also presented as a protective measure against the virus. I'm unsure about the safety of receiving the vaccine during pregnancy.”

Participant K, nearing her due date, sought clarity: “With my baby's development well underway, is it safe for me to receive the vaccine now, or should I wait until after the delivery and breastfeeding period?”

Participant L had doubts about vaccine coverage: “After receiving one dose of the COVID-19 vaccine, I'm left wondering whether it fully protects against COVID-19-related pneumonia.”

Participant M, contemplating the need for a complete vaccination series, asked: “I had two doses before getting pregnant and didn't complete the third dose before conceiving. Is my vaccination still effective? And is completing the third dose crucial for ongoing protection?”

Here, we observed a clear division in attitudes toward hospital visits for maternity check-ups. Some participants, such as Participant B, avoided hospital visits due to concerns about insufficient sanitation and the risk of exposure in crowded settings. Others, however, despite sharing these concerns, continued to attend check-ups, emphasizing the importance of regular medical supervision for the baby's health. This divergence highlights a conflict between the perceived risk of exposure and the perceived necessity of prenatal care. Conflicting information and decision-making: participant I's confusion about the best actions to take due to conflicting information underscores a significant divergence in how participants processed and acted upon COVID-19-related health information. This variation suggests a need for clear, consistent, and trustworthy health communication tailored to pregnant women's concerns.

In addition, the participants' concerns also reflect divergent views on the efficacy and safety of the COVID-19 vaccine during pregnancy. For instance, Participant E's regret about receiving the vaccine close to conception due to advisories against pregnant women getting vaccinated highlights the confusion and mixed messages surrounding vaccination. Meanwhile, Participant L's uncertainty about the protection offered by a single vaccine dose points to a gap in understanding about vaccine efficacy and the importance of a complete series.

#### 3.2.2 Theme II: maternal health

##### 3.2.2.1 Nutrition and diet

Thirteen study participants, especially those in their first pregnancy, with unplanned pregnancies, experiencing severe early pregnancy symptoms, or concerned about excessive weight gain, expressed significant concerns about maintaining a healthy diet. They were in search of advice on appropriate dietary choices and what foods to avoid.

Participant A highlighted the challenge of balancing nutrition with early pregnancy symptoms: “Understanding the critical role of nutrition during pregnancy, I find it difficult to identify foods that are both nutritious and suitable for managing early pregnancy symptoms.”

Participant B, facing an unplanned pregnancy, sought guidance on nutrition: “Being unprepared due to my unplanned pregnancy, I am keen to learn what is best for the health of both myself and my baby, including how to maintain a healthy weight and ensure the baby's nutritional needs are met.”

Participant C, experiencing her first pregnancy, expressed concerns about weight management: “My family encourages me to eat more because it's my first pregnancy. However, I am worried about gaining too much weight and want to know how to balance getting enough nutrients without excessive weight gain.”

Participant D recognized the importance of dietary knowledge: “I know diet is important during pregnancy, but need information on specific foods that should be included or avoided.”

Participant E dealt with the impact of nausea on diet: “Dealing with constant nausea makes eating well difficult. I'm drawn to acidic foods like hawthorn (Hawthorn is a kind of sour fruit, and many pregnant women want to have sour food when they are pregnant and vomiting. Although food with sour flavor can relieve nausea and vomiting that occur in pregnant women, not all sour food is suitable for pregnant women to consume, and hawthorn is an exception. Hawthorn is an exception. Hawthorn has the effect of breaking blood and dispersing silt, which can stimulate uterine contraction and may induce miscarriage. Hawthorn contains a lot of fruit acids, which have the effect of astringency and stimulating the gastric mucosa. After pregnancy, pregnant women's spleen and stomach function is poor, eating more hawthorn is easy to reduce the digestive ability and cause indigestion) but am confused by mixed messages regarding their safety.”

Participant E also questioned the timing of folic acid intake: “I conceived unexpectedly and began folic acid late. Could this lead to developmental issues, as suggested online?”

Participant F emphasized the need for detailed guidance: “As this is my first pregnancy, I'm looking for detailed advice from healthcare providers on diet, exercise, and what symptoms to expect for a healthy pregnancy journey.”

Participant G discussed the need for a balanced diet: “It's vital for pregnant women to carefully manage their diet to ensure they're getting enough nutrients while avoiding too much caffeine, sugar, and other harmful substances. Following medical advice and regular check-ups are key.”

Participant H shared concerns about late folic acid supplementation: “This pregnancy was unexpected, and I started taking folic acid later than recommended. I'm worried about how this delay might affect my baby's development.”

Participant I focused on the importance of holistic health care: “Maintaining overall health through a balanced diet, sufficient rest, and regular exercise is essential for pregnant women.”

Participant J inquired about dietary management in late pregnancy: “As I approach the end of my pregnancy, I wonder if strict dietary control is still necessary.”

Participant K addressed the issue of weight management: “Finding a balance between the weight gain recommendations from doctors and family advice is difficult. I need guidance on how to provide the best nutrition for my baby without excessive weight gain.”

Participant L aimed for a balanced approach to health: “My main goal is to keep myself healthy while minimizing any negative impact on the fetus. I'm interested in learning about recommended dietary and exercise practices.”

Participant M desired comprehensive information: “I want to learn more about all aspects related to pregnancy, including diet, exercise, and mental health.”

The participants displayed varied strategies in managing nutritional intake, balancing between craving management and symptom alleviation. While some sought specific dietary advice to mitigate early pregnancy symptoms, others focused on weight management, indicating a spectrum of priorities and concerns related to diet. In addition, there was a clear divergence in approaches to weight management, with some participants concerned about excessive weight gain and seeking guidance to avoid it, while others felt pressure to eat more to support the baby's growth, reflecting varied familial and cultural influences on dietary practices during pregnancy.

##### 3.2.2.2 Exercise and rest

During our interviews, nine participants shared insights into their experiences with exercise during pregnancy. It was observed that the comfort level and ability to engage in physical activity varied significantly across different pregnancy stages. Women in their mid-pregnancy often found it easier to participate in exercise, whereas early pregnancy symptoms and the fear of inducing premature labor were common deterrents. As pregnancy progressed, the intensity and variety of exercises diminished, with walking emerging as the most favored form of physical activity.

Participant B discussed her exercise routine: “I've been engaging in prenatal yoga and fitness routines tailored for pregnant women. I feel more comfortable being active in my mid-pregnancy despite early pregnancy discomforts. Before I exercise, I always check online to ensure the activities are safe.”

Participant C, in her thirties and nearing the third trimester, expressed interest in expanding her exercise options beyond walking, seeking safe activities suitable for this later stage of pregnancy.

Participant E encountered challenges with staying active due to early pregnancy symptoms like nausea and vomiting, which sometimes made even walking difficult.

Participant F, experiencing pregnancy for the first time, mentioned her simple exercise routine: “I take walks after dinner but am uncertain about other exercises that would be safe for me.”

Participant G, an older first-time pregnant woman, highlighted the importance of caution and the value of walking as a shared activity with her husband, emphasizing the need for precise guidance on safe exercises from professionals.

Participant H preferred walking as her main form of exercise, consistent with her activities in previous pregnancies, though she noted that hot weather often deterred her from going outside.

Participant I adjusted her activities to accommodate her growing belly, opting for evening walks as a cautious exercise option.

Participant L engaged with online exercise programs, focusing on stretching and gentle movements, and intentionally avoided intense activities such as hiking and sports.

Participant M found a suitable exercise in yoga, attending classes led by an instructor to ensure fitness while being mindful of her body's post-pregnancy changes.

These accounts reflect the diverse approaches to exercise among pregnant women, underscoring the importance of safety, comfort, and personal preferences in maintaining physical activity during pregnancy. Overall, the comfort and ability to engage in exercise varied, with some participants integrating specific prenatal routines comfortably into their daily lives, while others found even mild exercise challenging due to symptoms or concerns about safety, indicating a need for personalized exercise guidance. Moreover, the degree of engagement in physical activities showed divergence, with some participants actively seeking out safe exercises and others defaulting to walking as a safe option, highlighting differences in personal initiative, perceived safety, and available resources for exercise during pregnancy.

##### 3.2.2.3 Sexual life

For this topic, discussions around sexual life during pregnancy revealed a notable reticence among participants, a reflection of broader cultural attitudes. Only a few women felt comfortable sharing their experiences, underscoring the sensitivity and privacy typically associated with this topic in Chinese culture. The primary concern for those who did speak on the matter was the safety of the fetus, influencing their decisions to engage in or abstain from sexual activity, especially during the early and late stages of pregnancy.

Participant B, drawing on her personal experience from a second pregnancy, noted that the middle trimester is often seen as a safer period for sexual activity. She emphasized the importance of proceeding with caution, acknowledging sexual intimacy as a part of life but one that requires careful consideration during pregnancy.

Participant L spoke openly about her experiences with intimacy, sharing that she and her husband had engaged in sexual activity with her husband on top, a position they found to be safe during what is considered a relatively safe month of pregnancy. Her openness highlighted a level of comfort and excitement in exploring intimacy within the perceived safety parameters of pregnancy.

Participant M, at 20 weeks pregnant, expressed a keen interest in exploring sexual intimacy discreetly with her husband. She valued conversations with individuals who had more experience in this area, indicating a desire for guidance and reassurance.

These accounts illustrate the varied approaches to navigating sexual intimacy during pregnancy among the participants. While some felt comfortable discussing and exploring this aspect of their lives, others preferred silence or abstention, guided by concerns for fetal health and influenced by cultural norms. These underscore the importance of providing personalized, flexible, and culturally sensitive health education and support to address the varied concerns and priorities of pregnant women effectively.

#### 3.2.3 Theme III: fetal health

##### 3.2.3.1 Medications and hazardous substances

Within the scope of concerns related to fetal health, some of our participants voiced apprehensions regarding the use of medications during pregnancy. The focus of their worry included antibiotics, exposure to chemicals, as well as the impact of smoking and alcohol consumption, all feared for their potential adverse effects on fetal development.

Participant D reflected on her actions before knowing she was pregnant, stating, “I was not aware of my pregnancy during the 1st to 2nd months, and during that time, I dyed my hair and frequently ate fast food. Now, I'm burdened with anxiety about how these actions may have affected my fetus.”

Participant H discussed the challenges posed by social obligations, “Due to my husband's work-related events, we often find ourselves in environments where smoking and drinking are prevalent. Discovering our unplanned pregnancy and with an NT scan [Nuchal Translucency scan] on the horizon, my concerns for the fetus's wellbeing have significantly heightened.”

Participant I recounted her dilemma with medication, “While we were planning for a pregnancy, I inadvertently took cold medicine shortly before learning I was pregnant. The uncertainty of how this might affect my baby's development is a constant worry, with the fear of potential abnormalities being particularly distressing.”

Here, we observed variance in participants' perceptions of risk regarding medication use and exposure to hazardous substances, with some expressing significant anxiety about accidental exposures early in pregnancy, while others were more concerned about social environments' impact on fetal health.

##### 3.2.3.2 Pregnancy check-ups

The topic of pregnancy check-ups emerged as a point of concern for several participants, who were eager for clarity on the procedures involved, the implications for fetal health, and the necessary precautions to be observed before and after these check-ups.

Participant A was curious about the protocols for assessing the risk of Down's syndrome, asking, “Why are there different recommendations from doctors for Down's syndrome testing—between screening and non-invasive DNA tests? How do these methods operate, and is there any risk to the baby associated with them?”

Participant H brought up questions about a specific diagnostic procedure, “I'm trying to understand amniocentesis better. It was never suggested in my earlier pregnancies. What exactly does it involve, is it considered safe for someone of my age, and what are the risks to the baby?”

Participant L expressed concerns related to the scheduling of ultrasound appointments amid the ongoing pandemic, “The epidemic has led to the rescheduling of maternity exams, including my 21-week check-up. This has left me wondering about the best timing for undergoing a 3D or 4D ultrasound.”

Participant M sought detailed information on fetal development assessments, “I'm curious about macrosomia and what it entails. Which specific aspects of fetal development are examined for potential abnormalities? Is it necessary to fast before undergoing such an exam, given that my baby tends to be more active when I feel full? Could this activity level influence the results of the ultrasound?”

Overall, the participants differed in their information needs regarding pregnancy check-ups, with some seeking detailed explanations of specific procedures and their implications, while others were more concerned about the logistical aspects of scheduling and attending check-ups during the pandemic.

##### 3.2.3.3 Fetal movement monitoring

Fetal movement is a significant indicator of a baby's wellbeing inside the womb, and it's understandable why it would be a source of concern for expecting mothers. The participants' experiences and questions highlight the need for clear, accessible information on what to expect and how to effectively monitor fetal movements.

Participant C's challenge with the impracticality of hourly movement counts due to a busy schedule is a common concern. Practical advice for busy mothers-to-be could involve setting aside specific times when they are more likely to notice movements, such as after meals or during periods of rest.

Participant F's experience of reduced fetal movement after overeating and the subsequent relief of finding everything was fine with her baby underlines the importance of awareness and prompt action when changes are noticed. It also points to the need for guidance on how normal physiological activities, like eating, can influence fetal movement.

Participant G's proactive approach, opting for an extra ultrasound after noticing changes in movement due to skipped meals, reflects the anxiety that can come with the responsibility of monitoring fetal health. This situation underscores the need for clear guidelines on when to seek medical advice and how maternal activities and behaviors can affect fetal movements.

Participant M's concern about not feeling fetal movements by 20 weeks and the resulting frequent hospital visits for reassurance speak to the anxiety first-time mothers, in particular, might experience. This highlights the necessity for education on the expected timeline for feeling fetal movements and when the lack of movement warrants medical attention.

Finally, Participant J's worry about decreased fetal movement as labor approaches and the reassurance received from a doctor touches on the need for continuous communication and reassurance from healthcare providers. It emphasizes the importance of understanding the normal changes in fetal movement patterns toward the end of pregnancy and when to seek help.

Overall, there was a range of practices and levels of concern regarding fetal movement monitoring. Some participants were proactive and sought additional check-ups upon noticing changes, while others experienced anxiety due to not feeling movements at expected times, highlighting varied responses to monitoring fetal health signs. These experiences point to a broader need for accessible, clear information and guidance for expectant mothers on monitoring fetal movements, understanding what is normal, and recognizing signs that may warrant further medical evaluation.

#### 3.2.4 Theme IV: knowledge of childbirth

This theme encompasses the participants' perspectives and concerns regarding childbirth, highlighting their preferences for family accompaniment, considerations about labor analgesia, and choices regarding the mode of delivery. Each subtheme reflects a blend of personal desires, medical considerations, and emotional responses to the prospect of childbirth.

##### 3.2.4.1 Family accompaniment

The desire for family support during childbirth was important for several participants, reflecting the emotional and psychological comfort it provides.

Participant C expressed a wish for her husband's presence to share in the birth experience.

Participant I hoped for her mother's support, emphasizing the value of maternal presence during such a significant life event.

Participant J, while valuing family support, acknowledged the critical role of medical staff in ensuring safety and wellbeing during childbirth.

Participant K offered a different perspective, preferring privacy over the presence of her husband during potentially messy childbirth moments.

Collectively, we observed that participants had divergent views on family support during childbirth, with some desiring the presence of their partner or mother for emotional support, while others preferred privacy, highlighting the personal nature of childbirth preferences.

##### 3.2.4.2 Analgesia in childbirth

The discussion around labor analgesia revealed a range of emotions, from fear and confusion to the desire for a pain-free experience.

Participant G discussed the dilemma between fearing labor pain and worrying about the potential impact of analgesia on the baby.

Participant I, with a previous positive experience with painless labor, was open to using analgesia again, depending on the situation.

Participant J sought clarity on the availability and safety of painless labor options across hospitals, reflecting common concerns among expectant mothers.

Participant K, influenced by her friends' experiences, prioritized pain reduction in her childbirth plan.

From these, it can be observed that attitudes toward labor analgesia varied significantly, from fear of its effects on the baby to a strong preference for pain reduction. This diversity underscores the complexity of decisions around pain management in childbirth.

##### 3.2.4.3 Choice of mode of delivery

The choice between vaginal birth and cesarean section was a topic of contemplation and concern for participants.

Participant G expressed a preference for vaginal birth but was wary of the potential for complications leading to emergency cesarean sections. She said “With the increasing prevalence of cesarean sections, I worry about unsuccessful attempts leading to further distress.”

Participant I felt encouraged by a smooth first delivery, hopeful for a similar experience the second time around, mentioning “A smooth first delivery suggests a smoother second one.”

Participant J, close to her due date, considered a cesarean section, influenced by fear of labor pain and the perceived control it offers over the birthing process. She revealed, “In my advanced stage, I'm unsure. I might defer to my doctor's recommendation, though I lean toward a cesarean due to fear of pain.”

Participant K aspired to a vaginal birth but was apprehensive about the associated pain. She mentioned “I aspire to a vaginal birth but dread the pain,” illustrating the complex considerations women navigate when deciding on their preferred mode of delivery.

Overall, the preferences for vaginal birth vs. cesarean section were mixed, influenced by previous experiences, fears of labor pain, and perceptions of control over the birthing process. This variation illustrates the personal and complex nature of deciding on a mode of delivery.

#### 3.2.5 Theme V: knowledge of postnatal recovery

This part captures the concerns and anticipations of seven mothers regarding their recovery after childbirth. The focus on body image, abdominal skin recovery, and pelvic floor muscle strength reflects a blend of personal health priorities and societal influences. Traditional practices and cultural beliefs, such as the “sitting in the moonlight” period, play a significant role in shaping their recovery expectations and timelines.

Participant A worries about the impact of age on her postnatal recovery, highlighting a common concern that recovery might be more difficult for older mothers. This reflects a broader societal perception about the challenges of post-pregnancy body changes and weight loss in older women.

Participant D's experience brings attention to the physical and emotional impact of visible post-pregnancy changes, such as a prominent stomach and stretch marks, which can lead to uncomfortable social interactions and concerns about physical appearance.

Participant E, planning for weight loss after her third child, indicates a proactive approach to postnatal recovery, recognizing the importance of dedicating time and effort to regain pre-pregnancy body weight and shape.

Participant H's mention of temporary urinary incontinence after her second childbirth emphasizes the practical health concerns associated with pelvic floor recovery. Her commitment to rehabilitation post-birth underscores the importance of addressing these issues to improve quality of life and physical wellbeing.

Participant J's plan to rest for a month before focusing on weight loss suggests a balanced approach to recovery, acknowledging the need for physical rest and a gradual return to pre-pregnancy fitness and body shape.

Participant L's comment on the perceived benefits of early childbirth on recovery speed reflects cultural and societal beliefs about age and postnatal recovery. This belief may influence younger mothers' expectations and concerns about their post-pregnancy bodies.

Participant M's curiosity about specific postpartum changes, such as buttock enlargement and urinary leakage, highlights the desire for more comprehensive information on the physical recovery process. This interest in understanding the nuances of postnatal changes suggests a proactive approach to managing and mitigating these issues.

Collectively, these reflections underscore the complexity of postnatal recovery, encompassing physical, emotional, and societal dimensions. The mothers' concerns and plans reveal a deep-seated need for support, information, and practical strategies to navigate the postpartum period effectively.

#### 3.2.6 Theme VI: sources of knowledge on health education for pregnant women and their expectations of healthcare providers

Participants in the study reported using a variety of sources for health education during pregnancy, including the Internet (via platforms like Baidu and mother-and-baby applications), WeChat groups, the experiences of friends, and the advice of healthcare professionals. They often combined these methods to gather necessary information, with thirteen participants particularly favoring online resources.

Participant A described her method: “During my first pregnancy, I used Baidu for dietary questions, adhered to the advice found in books, and consistently discussed any concerns with medical staff during check-ups.”

Participant B preferred direct consultation with healthcare experts: “My main sources are doctors and nurses. The epidemic makes hospital visits difficult, so I also participate in WeChat groups for updates, even though these discussions sometimes miss professional insights.”

Participant C highlighted the utility of digital applications: “I use a pregnancy app for intermittent insights and confirm its advice with doctors. Remote consultations would be a beneficial option under the current health advisories.”

Participant D suggested the importance of professional guidance in online forums: “I participate in WeChat groups for expectant mothers, recommending these forums host discussions led by healthcare experts.”

Participant E utilized a mix of past experiences and digital tools: “With two pregnancies behind me, I rely on my own experiences and conversations with friends who are pregnant, along with updates from a pregnancy app named ‘Beauty Grapefruit.”'

Participant F mentioned the logistical challenges of attending hospital classes: “Hospital maternity classes are useful but scheduling conflicts arise. Having the option to replay these classes would aid in learning.”

Participant G described a proactive strategy: “I independently researched and read literature on pregnancy, supplementing this with insights from friends and family.”

Participant H used a blend of historical knowledge and current discussions: “I base my understanding on previous pregnancies and enhance it with current group discussions.”

Participant I engaged with local support networks: “I join a local WeChat group of pregnant women for active discussions on health topics. My attendance at in-person classes is sporadic due to the epidemic.”

Participant J sought information online: “I use the internet to investigate practices related to pregnancy.”

Participant K called for clear communication from healthcare providers: “It's crucial that healthcare providers offer detailed, patient-focused explanations. Online platforms could help address our questions more efficiently, considering their tight schedules.”

Participant L adopted a multifaceted approach: “Being my first pregnancy, I extensively read and seek out online consultations.”

Participant M interacted within digital communities cautiously: “I participate in WeChat discussions, but direct responses from doctors are rare due to their obligations. I limit my interactions to reduce any risk of complications.”

Overall, this theme illustrates the diverse and adaptive strategies pregnant women employ to educate themselves about health during pregnancy and their expectation for accessible and clear communication from healthcare providers.

## 4 Discussion

### 4.1 Personal protection and vaccine safety

This study highlights the measures Chinese pregnant women undertake to protect themselves and their unborn children amidst public health crises, such as the COVID-19 pandemic. Participants exhibited a readiness to self-isolate and curtail routine pregnancy check-ups, motivated by the objective to avert potential infections. Nonetheless, it is crucial to acknowledge that excessive anxiety during epidemics can lead to negative outcomes, as highlighted in prior research (21). The Chinese government has recommended a prudent approach, advising pregnant women to minimize non-essential medical visits while emphasizing the importance of attending essential prenatal examinations (22). Furthermore, healthcare facilities have adopted stringent infection control protocols, including facility disinfection, the provision of one-on-one consultations, and the enforcement of social distancing during wait times (23) Media and internet resources have been instrumental in communicating protective strategies, thus reducing the anxiety of pregnant women before consultations and enhancing their safety during medical visits (17).

Moreover, participants expressed significant concerns regarding the safety and efficacy of the COVID-19 vaccine. Given their dual role as healthcare recipients and guardians of their own and their fetuses' wellbeing, they sought affirmation on the vaccine's appropriateness. Although public health guidelines such as social distancing and mask usage are critical, the introduction of the COVID-19 vaccine presented an additional layer of preventive measures (24, 25). Initially, the National Health and Wellness Commission advised against administering the COVID-19 vaccine to women of childbearing potential and those breastfeeding. However, subsequent studies have validated the vaccine's safety and efficacy for pregnant individuals (26–29). Research indicates that COVID-19 vaccination does not increase the likelihood of teratogenic effects, congenital anomalies, spontaneous abortions, or low birth weights in comparison to those who are unvaccinated (*p* < 0.05).

### 4.2 Knowledge needs of pregnant women and fetal health

This study highlights the critical need to address the knowledge gaps among pregnant women regarding dietary practices essential for fetal development. Participants expressed a strong desire for information on nutritious dietary choices, underscoring the need for comprehensive and actionable counseling from healthcare providers. Such counseling should provide clear guidelines on prenatal nutrition, focusing on the consumption of nutrient-dense foods, the avoidance of potentially harmful substances, and methods to alleviate common pregnancy-related discomforts.

Collaboration between healthcare providers and dietitians is essential in delivering accurate information and personalized dietary advice that meets individual preferences and needs. Given the unique challenges and uncertainties presented by public health crises, healthcare professionals have a significant duty to educate and support pregnant women in making informed dietary decisions that promote maternal and fetal health. Proper nutrition during pregnancy is vital for the health of both the mother and the fetus (30). By offering evidence-based guidance, healthcare providers can enable pregnant women to make wise dietary choices, thus improving health outcomes.

During public health emergencies, the mental health of pregnant women has become a critical concern (31). Insights from interviews conducted in this study indicate that participants experienced increased stress, anxiety, and emotional instability, particularly highlighted during the COVID-19 pandemic. It is essential to develop targeted mental health resources and support mechanisms to assist pregnant women in effectively managing these challenges. Initiatives should include stress management techniques, counseling services, and awareness campaigns about available support networks. The integration of mental health support into routine prenatal care is crucial for addressing the psychological wellbeing of pregnant women during public health crises (31). This approach should include systematic screening for mental health issues, offering counseling services, and ensuring timely referrals to specialized resources. Evidence-based interventions, such as the Centering Pregnancy (CP) model of care, positive cognitive exercises, and peer support, have shown effectiveness in enhancing emotional wellbeing during pregnancy (32–36). CP originated in the United States (36) and consists of three main components, as follows: (1) Creation of a discussion group: 8–12 pregnant women of similar gestational weeks are divided into a group, and each group is led by a doctor and a midwife, respectively (the families of the pregnant women may also participate). (2) Pregnancy self-management: doctors or midwives conduct one-on-one antenatal check-ups for pregnant women. Pregnant women are instructed and encouraged to complete simple examinations such as weight, blood pressure, abdominal circumference, and fetal heartbeat monitoring on their own and record them. After the check-up, the pregnant woman can consult the midwife about her problems. If it is a common problem during pregnancy, the midwife encourages her to solve it together as a group. Between examinations, pregnant women can communicate and discuss freely. (3) Group discussion: starting from 12 to 16 weeks, group discussion is organized on a regular basis, 0.5–2 h each time for a total of 10 sessions. Pregnant women are encouraged to speak and discuss, share their feelings and experiences, guide the content of the discussion, and answer the questions raised by the pregnant women. Currently, CP is widely used in Western countries, including North America, Australia, the United Kingdom, and Africa (37–39), and it is agreed that CP is the best model of maternal health care, which promotes active participation of pregnant women, improves their mental health, and enhances satisfaction with care, prenatal knowledge, and ownership of self-care; and reduces the risk of preterm labor and cesarean section (40–43), while in China, the use of CP is still in its infancy. In China, the use of CP is still in its infancy, and some studies have found that CP can reduce pregnant women's fear of labor, enhance their breastfeeding self-efficacy (44, 45), promote comprehensive support from midwives, family members, and peers at the same time, and improve coping with perinatal problems (46). Mindfulness is the ability to describe an individual's awareness of being in the present moment. The level of mindfulness can be a useful predictor of depression to some extent, with higher levels of mindfulness being associated with lower levels of depression (33). When the level of positive thinking of pregnant women is low, they are unable to focus on the present moment and frequently focus on negative life events during pregnancy or after delivery, which may cause them to have negative emotions such as irritability and depression. Research has shown that a certain period of positive thinking training can improve the participants' positive thinking level and negative emotions (33). Currently, the most important positive thinking interventions include positive thinking stress reduction therapy and positive thinking cognitive behavioral therapy (33), which can reduce the generation of negative emotions, improve the quality of life, and promote the emotional stability of the mind during pregnancy and postpartum care.

### 4.3 Improving health education and guidance during pregnancy

Public health emergencies can restrict pregnant women's access to routine antenatal screening services due to hospital limitations and concerns for the safety of both the women and their fetuses. These restrictions may affect the detection of pregnancy complications and their overall management. To maintain continuous care, healthcare systems must adopt innovative methods for providing remote prenatal monitoring, health education, and guidance, such as through telemedicine and virtual counseling. Since the beginning of the COVID-19 pandemic in 2019, there has been a notable increase in pregnant women seeking personal protection against the novel coronavirus infection during pregnancy and childbirth (9). Furthermore, about 71.0% of Chinese pregnant women have turned to health apps, official microblogs, and WeChat public channels for information, highlighting the Internet's critical role during public health crises as an essential resource for pregnant women to obtain pregnancy-related information (47, 48).

The study revealed that participants predominantly utilized internet searches, pregnancy health applications, WeChat groups, and public forums for acquiring health-related information during public health emergencies. Nonetheless, skepticism concerning the expertise of information sources, including biomedical professionals, led to pregnant women harboring reservations about the reliability of health knowledge obtained online. They exhibited a greater trust in health education and information disseminated by certified biomedical practitioners. It is observed that medical professionals participating in group chats, such as on WeChat, may find it challenging to meet the personalized health education needs of each pregnant woman effectively. While telemedicine serves as a feasible option for certain prenatal care aspects, it faces limitations, such as the incapacity to perform specific physical examinations or tests remotely (49). As healthcare systems endeavor to provide continuous care amidst public health crises, it is imperative for providers, community-based organizations, and policymakers to develop and advocate for virtual support groups, online forums, and educational resources specifically designed for the needs of pregnant women. These initiatives can cultivate a sense of community, offer emotional support, and ensure access to trustworthy information, thereby mitigating isolation and improving overall wellbeing. Therefore, establishing robust communication channels between healthcare providers and pregnant women is essential for conveying accurate information, addressing concerns, and enabling shared decision-making.

### 4.4 Limitations

This study acknowledges the potential influence of social desirability bias on our findings, given the sensitive nature of the topics discussed. While measures were taken to ensure a comfortable and confidential environment for participants, the possibility that respondents might tailor their answers to what they perceive as more socially acceptable cannot be entirely ruled out. This bias could affect the authenticity and depth of the data collected, as participants might underreport or alter their experiences and needs. Additionally, its qualitative approach, while insightful, limits the ability to quantify the prevalence of specific needs or opinions, as it lacks quantitative data. This restricts the generalizability of findings and the measurement of opinion distribution among the broader population. Future research could benefit from employing mixed methods or anonymous data collection techniques to mitigate this limitation and capture a more authentic range of responses.

## 5 Conclusion

This study sheds light on the critical health education needs of pregnant women during public health emergencies during the COVID-19 pandemic. The empirical evidence provided serves as a valuable foundation, offering guidance for health education practitioners to develop targeted and impactful educational content. Nevertheless, this study is constrained by its limited sample size, the geographical limitation of the sample, and the potential for sample selection bias. To strengthen these findings, future research could include a broader and more diverse participant base and employ a variety of data collection techniques. In summary, this research emphasizes the vital role of health education for pregnant women during the COVID-19 pandemic, providing meaningful insights for healthcare providers and setting a direction for future investigations in this field. The insights from this pandemic highlight the importance of encouraging pregnant women to attend maternity examinations in a timely manner and to utilize biomedical resources wisely, ensuring the health and safety of both mothers and infants during public health challenges. By adopting comprehensive safety measures and isolation protocols, access to medical care for pregnant women can be improved, and leveraging internet resources can effectively promote the dissemination of healthcare knowledge related to pregnancy.

## Data availability statement

The original contributions presented in the study are included in the article/supplementary material, further inquiries can be directed to the corresponding author.

## Ethics statement

The studies involving human participants were reviewed and approved by the Research Ethics Review Committee of Ethics Committee of Quanzhou Medical College (the code of ethics: No. 2021007). The studies were conducted in accordance with the local legislation and institutional requirements. The participants provided their written informed consent to participate in this study. Written informed consent was obtained from the individual(s) for the publication of any potentially identifiable images or data included in this article.

## Author contributions

XS: Data curation, Formal analysis, Funding acquisition, Investigation, Methodology, Project administration, Supervision, Writing – original draft, Writing – review & editing. YZ: Data curation, Funding acquisition, Methodology, Project administration, Resources, Supervision, Visualization, Writing – review & editing, Conceptualization. MC: Data curation, Formal analysis, Funding acquisition, Investigation, Project administration, Resources, Validation, Visualization, Writing – review & editing. XX: Data curation, Formal analysis, Investigation, Software, Writing – review & editing, Resources. GL: Conceptualization, Data curation, Methodology, Project administration, Supervision, Validation, Visualization, Writing – review & editing.
